# Supplementary figures and images for: Modeling relaxation experiments with a mechanistic model of gene expression
Source: BMC Bioinformatics. 2024 Aug 20;25:270. doi: 10.1186/s12859-024-05816-4 (PMC11334594; doi:10.1186/s12859-024-05816-4)

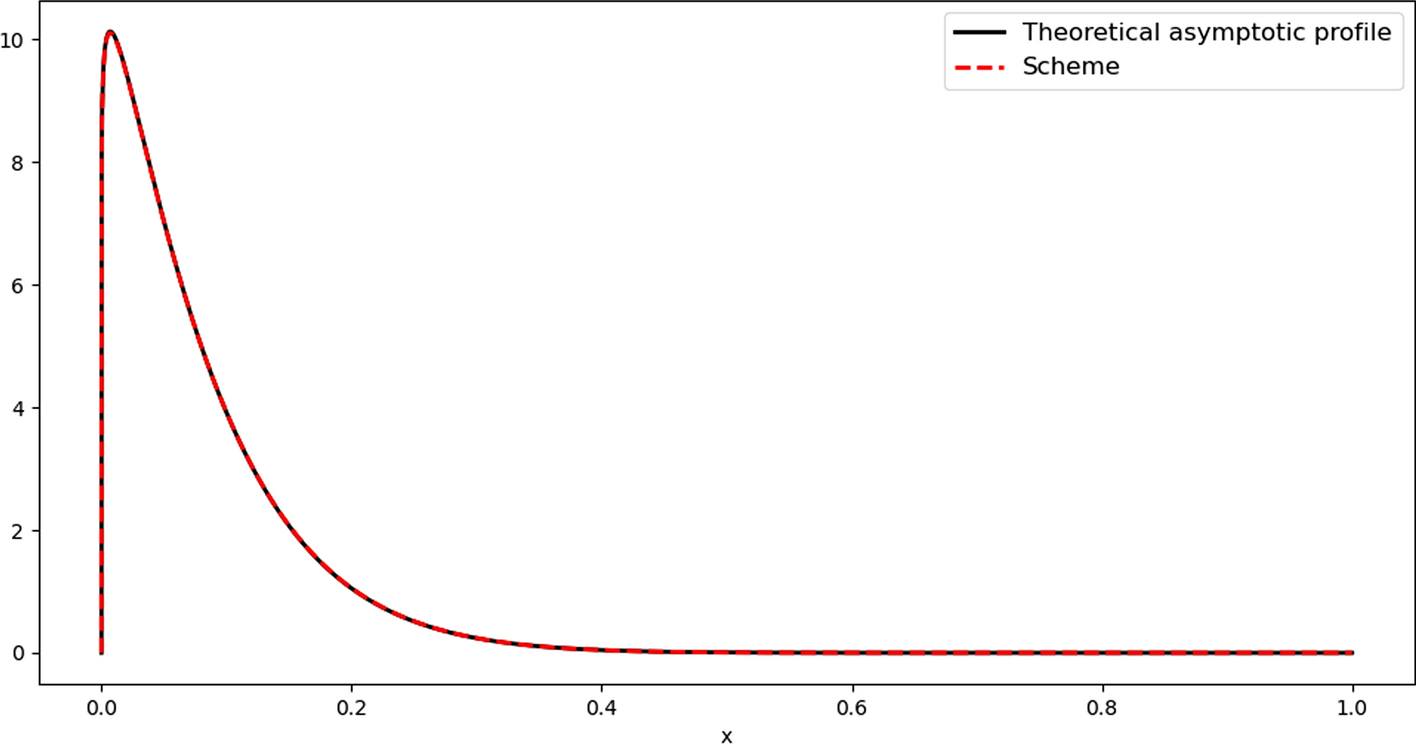

Supplement: Supplementary file 1 — Comparison between the theoretical asymptotic profile N (black line) presented in Theorem 1 and the numerical solution obtained using the numerical scheme (red dotted line) defined by Eq. (9). Parameter values are given by $r_0 = 3.4$r0=3.4, $r_1 = 3$r1=3, $k_{\text {on}} = 0.85$kon=0.85, $k_{\text {off}} = 12.71$koff=12.71, $d=1$d=1, $X_{\max }=2 \times 10^4$Xmax=2×10^4^ [file 12859_2024_5816_Fig7_HTML.png]
